# Enjoyment as a motivational driver of sport continuation: a moderated mediation model of engagement and social support

**DOI:** 10.3389/fpsyg.2026.1813793

**Published:** 2026-05-07

**Authors:** Zülbiye Kaçay, Emrah Seçer, Nuh Osman Yıldız, Oğuz Kaan Esentürk, Laurentiu-Gabriel Talaghir, Cristina Corina Bentea, Paula Ivan, Florentina Cristea

**Affiliations:** 1 Faculty of Sports Sciences, Çanakkale Onsekiz Mart University, Çanakkale, Türkiye; 2 Faculty of Sports Sciences, Erzincan Binali Yıldırım University, Erzincan, Türkiye; 3 Faculty of Sports Sciences, Bolu Abant Izzet Baysal University, Bolu, Türkiye; 4 Faculty of Physical Education and Sport, “Dunarea de Jos” University of Galati, Galati, Romania

**Keywords:** enjoyment of physical activity, moderated mediation model, perceived social support, sport engagement, sport participation continuity

## Abstract

**Introduction and aim:**

The present study aimed to examine the effect of enjoyment of physical activities on intention to continue sport participation and to determine the mediating role of sport engagement and the moderating role of perceived social support in this relationship.

**Methods:**

This correlational study included 748 athletes (445 males, 303 females, *M* = 23.10, SD = 5.59). The Physical Activity Enjoyment Scale, Intention to Continue Sport Scale, Sport Engagement Scale, and Perceived Social Support Scale–Short Form were used as data collection instruments. The measurement model and construct validity of the scales were tested using confirmatory factor analysis (AMOS 21). Hypotheses were tested in SPSS 25 using PROCESS Macro models (4, 1, and 14).

**Results:**

To test the first hypothesis, the mediation effect was examined using PROCESS Model 4 (PACES → SES → ICSS). The indirect effect of PACES on ICSS via SES was found to be significant (*b* = 0.230, 95% CI [0.184, 0.279]). For the second hypothesis, PROCESS Model 1 was applied. The main effect of perceived social support (PSS) was not significant (*b* = 0.076, *p* > .05), whereas the interaction term representing the SES × PSS interaction was significant (*b* = −0.0147, 95% CI [−0.0245, −0.0049]), indicating that perceived social support moderated the relationship between sport engagement and intention to continue sport participation. To test the third hypothesis, a moderated mediation model (PROCESS Model 14) was conducted with PSS as a moderator. The index of moderated mediation was significant (*b* = −0.010, 95% CI [−0.020, −0.0005]), showing that perceived social support moderated the indirect effect of PACES on ICSS through SES.

**Conclusion:**

The findings revealed that the indirect effect of enjoyment of physical activities on intention to continue sport participation through sport engagement was stronger among athletes with lower perceived social support.

## Introduction

Athletes’ continuation in sport is critically important for the sustainable development of performance and the maintenance of healthy lifestyle behaviors (Scanlan et al., 1993; Hodge et al., 2023). However, maintaining participation in sport cannot be explained solely by physical competence. Rather, it represents a multidimensional process shaped by individuals’ subjective pleasure derived from sport experiences, their psychological attachment to sport, and the support they perceive from their social environment (Ryan and Deci, 2020; Sallis et al., 2015). Therefore, psychological approaches grounded in intrinsic motivation have become increasingly prominent in explaining sustained sport participation (Deci and Ryan, 2000; Vasconcellos et al., 2020). Enjoyment of physical activities, as a central component of intrinsic motivation, is considered an important factor that strengthens individuals’ tendency to continue engaging in sport voluntarily. Enjoyment supports the intention to continue sport participation by associating sport experiences with positive emotions. Nevertheless, the psychological mechanisms through which this effect emerges have been addressed only to a limited extent in literature. In this regard, sport engagement—reflecting athletes’ commitment-like psychological involvement in sport—emerges as a functional mediating mechanism through which enjoyment derived from physical activities may translate into a stronger intention to continue sport participation. Moreover, sport behavior is shaped not only by individual psychological processes but also by the broader social context. Perceived social support may strengthen or weaken the effect of sport engagement on intention to continue sport participation by influencing athletes’ motivational processes. In the present study, enjoyment of physical activities, sport engagement, and perceived social support are examined simultaneously within a model aiming to explain athletes’ intention to continue sport participation. Taken together, Self-Determination Theory explains the intrinsic motivational basis of enjoyment, while the Theory of Planned Behavior provides a framework for understanding how engagement contributes to intention formation. In addition, Social Cognitive Theory and Ecological Systems Theory highlight the role of social support as a contextual factor shaping behavioral outcomes. Therefore, the present study integrates these complementary theoretical perspectives into a unified model to explain how enjoyment translates into intention to continue sport participation through engagement, and under which social conditions this process becomes stronger or weaker.

### Enjoyment of physical activities and intention to continue sport participation

Enjoyment derived from physical activities is widely recognized as one of the core psychological factors that nurtures individuals’ intrinsic motivation toward sport and plays a determining role in the sustainability of sport participation. According to Self-Determination Theory (SDT), individuals’ tendency to maintain an activity is directly associated with the pleasure and satisfaction experienced during that activity. In particular, intrinsic motivation (Frikha et al., 2024) and self-regulatory forms of motivation strongly shape the long-term maintenance of behavior (Deci and Ryan, 2000). In addition, individuals who engage in physical activity with intrinsic motivation tend to experience higher levels of enjoyment and greater life satisfaction (Seçer and Bayrakdar, 2025). This is suggested to significantly influence the continuity of physical activity behaviors (Calder et al., 2020). Similarly, a positive relationship has been reported between the level of enjoyment derived from physical activity and habit formation, emphasizing that enjoyment contributes to sustained participation in physical activities (Weyland et al., 2024). Enjoyment of physical activities enhances individuals’ mental wellbeing and strengthens their intention to continue sport participation by transforming sport into a meaningful and autonomous experience (Shan et al., 2025; Zekioğlu et al., 2024). Expectancy–Value Theory provides a complementary framework to explain this relationship. According to this theory, individuals’ intention to maintain a behavior is shaped by the outcomes they expect to obtain and the value they attribute to that behavior. Moreover, perceiving an activity as enjoyable becomes a strong determinant of behavioral intention (Eccles and Wigfield, 2002). Accordingly, the effect of enjoyment of physical activities on intention to continue sport participation is strongly supported by both SDT and Expectancy–Value Theory perspectives. While SDT explains individuals’ intrinsic motivation, Expectancy–Value Theory clarifies the role of enjoyment and value perceptions on intention. Taken together, these two theories provide a comprehensive explanation of the psychological mechanisms underlying intention to continue sport participation.

### The mediating role of sport engagement

Sport engagement refers to individuals’ sustained, energetic, and dedicated involvement in sport activities and is considered a key psychological mechanism supporting continued participation (Guillén and Martınez-Alvarado, 2014; Kayhan et al., 2020). In the sport context, engagement reflects a persistent motivational state characterized by vigor and absorption, which is closely linked to athletes’ willingness to maintain sport participation over time.

From a cognitive perspective, the Theory of Planned Behavior (TPB) proposes that attitudes, subjective norms, and perceived behavioral control contribute to individuals’ intention to continue sport participation (Ajzen, 1991; Cunningham and Kwon, 2003). Within this framework, engagement can be viewed as a motivational state that strengthens intention formation by fostering positive evaluations of sport participation and reinforcing volitional control. Likewise, from the perspective of Self-Determination Theory (SDT), higher levels of autonomous motivation and satisfaction of basic psychological needs are expected to promote engagement in sport activities and facilitate the development of stable behavioral intentions (Deci and Ryan, 2000).

In addition, social-contextual approaches suggest that supportive environments may strengthen athletes’ engagement levels and enhance their intention to continue sport participation by reinforcing psychological involvement in sport settings (Hodge et al., 2023; Tao and Yu, 2025). In this respect, engagement may be considered conceptually close to commitment-related motivational processes that have been emphasized in the sport psychology literature as key predictors of sustained sport participation (Scanlan et al., 1993; Çaglar and Merdan, 2022).

### The moderating role of perceived social support

Perceived social support refers to individuals’ subjective evaluation of emotional, informational, and functional support received from their social environment (e.g., family, friends, peers, educators, coaches). In physical activity and sport continuation behaviors, social support functions both as a factor that directly encourages behavior and as a moderator that facilitates maintenance by strengthening psychological resources (Xu et al., 2024). Recent evidence has also shown a meaningful association between individuals’ physical activity levels and perceived social support, further emphasizing the role of supportive social contexts in sustaining engagement in sport and exercise (Özsaydı and Gorucu, 2025). According to Social Support Theory, support received from the social environment enhances individuals’ psychological resilience and motivational resources, thereby increasing their intention to continue engaging in activities. Specifically in the context of physical activity, types of social support such as peer and family support have been reported to enhance participants’ perceived self-efficacy, which in turn positively influences intention to continue sport participation (Xu et al., 2024; Zhou et al., 2025). Ecological Systems Theory (Bronfenbrenner, 1979) posits that individuals’ behaviors are shaped by the interaction of multilevel environmental systems. From this perspective, social support operates across interconnected contexts (e.g., family, peers, schools, and broader cultural structures) to facilitate sport participation and strengthen behavioral intentions (Bronfenbrenner, 1979; Sallis et al., 2015). According to Social Cognitive Theory, individual behaviors are shaped by the reciprocal interaction of environmental and psychological factors. Social support is described as a key environmental input that increases intention to continue sport participation by strengthening individuals’ perceived self-efficacy. Self-efficacy represents one’s belief in approaching tasks and coping with challenges, and social support reinforces this belief through mechanisms such as positive feedback, modeling, and confidence-enhancing processes (Bandura, 1986; Xu et al., 2024). Recent studies have also demonstrated that sport participation and social support are linked through a chain mediation effect, further confirming the importance of social support for behavioral outcomes (Tang et al., 2025). In this context, perceived social support is important not only for strengthening the relationship between sport engagement and intention to continue sport participation, but also as a moderating factor indicating that this relationship may vary depending on situational conditions. That is, the effect of sport engagement on intention to continue sport participation may differ across varying levels of social support. Therefore, perceived social support is addressed in this study as a critical moderating variable shaping the interactions of psychological and behavioral processes within the proposed model.

This study aimed to examine the effect of athletes’ enjoyment of physical activities on their intention to continue sport participation and to investigate the mediating role of sport engagement and the moderating role of perceived social support in this relationship. Accordingly, the hypotheses of the study were formulated as follows:

H1: Enjoyment of physical activities positively predicts intention to continue sport participation through its indirect effect via sport engagement.

H2: Perceived social support moderates the relationship between sport engagement and intention to continue sport participation, such that the positive association is stronger at lower levels of perceived social support.

H3: Perceived social support moderates the indirect relationship between enjoyment of physical activities and intention to continue sport participation via sport engagement, with the indirect effect being stronger at lower levels of perceived social support.

## Materials and methods

### Research design

**FIGURE 1:**
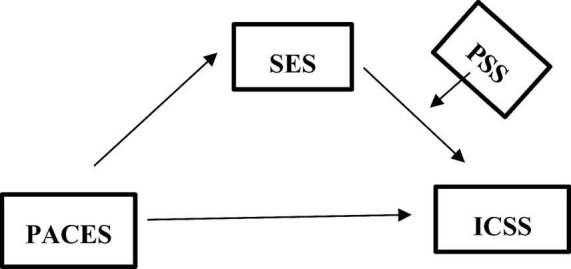
Conceptual model illustrating the mediating role of sport engagement and the moderating role of perceived social support.

This study aimed to examine the effect of athletes’ enjoyment of physical activities on their intention to continue sport participation. In addition, the mediating role of sport engagement and the moderating effect of perceived social support in this relationship were also addressed. The study was designed within the framework of the correlational survey model, which is one of the quantitative research approaches used to identify relationships between two or more variables (Christensen et al., 2015). In recent years, research in the social sciences has increasingly emphasized that identifying only direct relationships or unidirectional effects between variables may remain insufficient to explain the multidimensional and complex nature of social phenomena. Accordingly, it has been highlighted that moderator effects, which focus on the conditions under which relationships become stronger or weaker, and mediator effects, which explain the processes and mechanisms through which these relationships occur, should be examined together. Such analyses are considered to enable a more comprehensive testing of theoretical models and to provide original contributions to the field (Gürbüz, 2021a). The theoretical model tested in the present study is presented in Figure 1.

### Research group

Sampling was carried out using criterion sampling, which is one of the purposive sampling methods. This approach is based on constructing the sample from individuals, events, or situations that meet predetermined characteristics directly related to the research problem (Büyüköztürk et al., 2024). It has been emphasized that determining criteria in a manner consistent with the overall aim and theoretical framework of the study is methodologically critical (Creswell and Clark, 2017). Accordingly, the inclusion criteria for participation were defined as follows: (a) actively engaging in sport, (b) voluntarily participating in the study, and (c) being 18 years of age or older. A total of 809 athletes from various individual and team sport branches, primarily competing at the amateur level, participated in the study. However, after excluding forms containing erroneous, incomplete, or outlier data, the analyses were conducted with 748 athletes. The adequacy of the sample size was evaluated based on both theoretical and statistical criteria. Considering the minimum recommended sample size of 384 participants based on a ± 0.05 sampling error and a 95% confidence level, the present sample was found to more than sufficiently meet this requirement. In addition, a power analysis conducted using G*Power software (F tests, linear multiple regression: fixed model, R^2^ increase; f^2^ = 0.08; α = 0.05; statistical power = 0.95; number of predictors = 3) indicated that at least 219 participants would be sufficient. In this context, the sample consisting of 748 athletes was evaluated as highly robust and reliable in terms of both statistical power and representativeness. Accordingly, the general characteristics of the athletes in the study were as follows: 303 females (age range 18–40 years, 23.33 ± 5.382) and 445 males (age range 18–40 years, 22.94 ± 5.738). All participants were actively engaged in organized sport settings at the time of data collection.

### Procedure

Data were collected from athletes who voluntarily agreed to participate in the study. Prior to data collection, participants were informed about the purpose of the study, confidentiality, and their right to withdraw at any time. The questionnaires were administered in organized sport settings under the supervision of researchers. The data collection process was conducted in accordance with ethical standards, and all responses were obtained through self-report forms.

### Data collection tools

#### Physical Activity Enjoyment Scale (PACES)

The scale was originally developed by Mullen et al. (2011), and its Turkish adaptation was conducted by Özkurt et al. (2022). The scale consists of 8 items, is unidimensional, and is rated on a seven-point Likert-type scale. The scale includes no reverse-coded items, and evaluation is based on the total score. The possible scores range from 8 to 56, and higher total scores indicate a higher level of enjoyment derived from physical activity. In the Turkish adaptation study, the internal consistency reliability of the scale was found to be very high, and the Cronbach’s alpha coefficient was reported as .955. In addition, confirmatory factor analysis results demonstrated that the one-factor structure of the scale showed good fit. The reported fit indices were CMIN/DF = 2.368, GFI = 0.98, CFI = 0.99, TLI = 0.99, RMSEA = 0.042, and SRMR = 0.010.


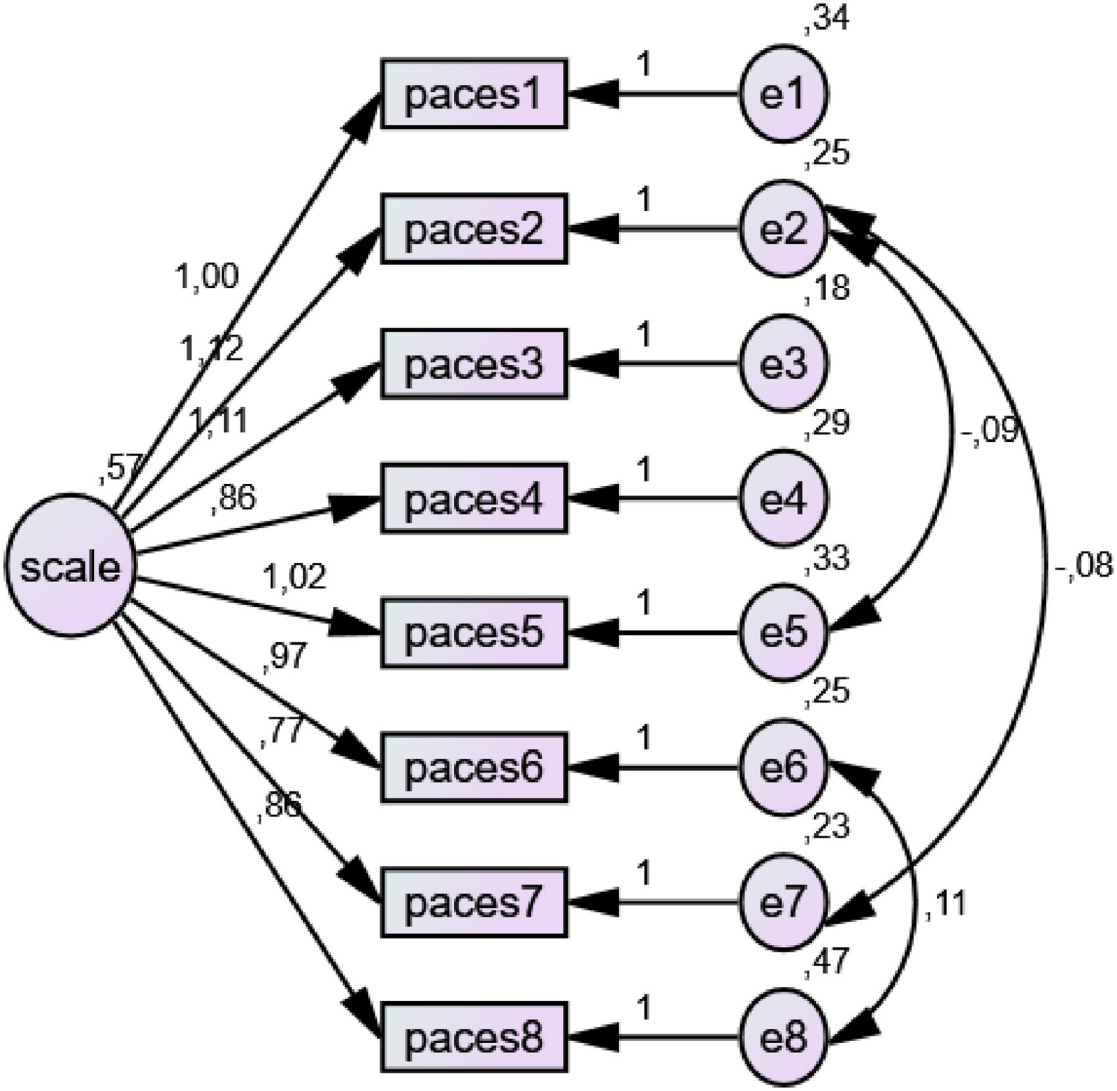


#### Intention to Continue Sport Scale (ICSS)

The scale was developed by Özkurt et al. (2025) and has a unidimensional structure consisting of six items. It is rated on a five-point Likert-type scale, with total scores ranging from 6 to 30. Higher scores indicate a stronger intention to continue sport participation. The scale includes no reverse-coded items. In the scale development study, internal consistency reliability was found to be very high, and Cronbach’s alpha coefficient was calculated as 0.98. Confirmatory factor analysis results indicated that the one-factor structure of the scale provided an acceptable level of model fit. The reported fit indices were CMIN/DF = 3.04, GFI = 0.96, CFI = 0.99, and RMSEA = 0.071.


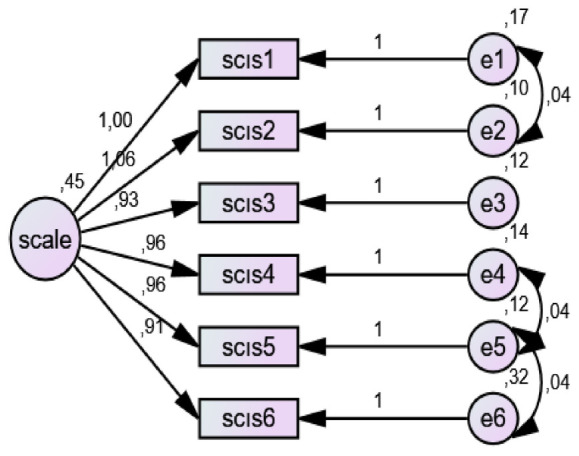


#### Sport Engagement Scale (SES)

The scale was developed by Guillén and Martınez-Alvarado (2014), and its Turkish adaptation was conducted by Kayhan et al. (2020). In its Turkish version, the scale consists of 10 items and two subdimensions (Vigor and Absorption), rated on a seven-point Likert-type scale (1 = Hardly ever, 7 = Almost always). The scale contains no reverse-coded items. The internal consistency reliability of the overall scale was calculated as Cronbach’s α = 0.918, while the Cronbach’s α coefficients for the subdimensions were 0.916 and 0.778, respectively. Confirmatory factor analysis results supported the structural integrity of the scale, and the reported fit indices were CMIN/DF = 1.63, GFI = 0.93, CFI = 0.96, and RMSEA = 0.05.


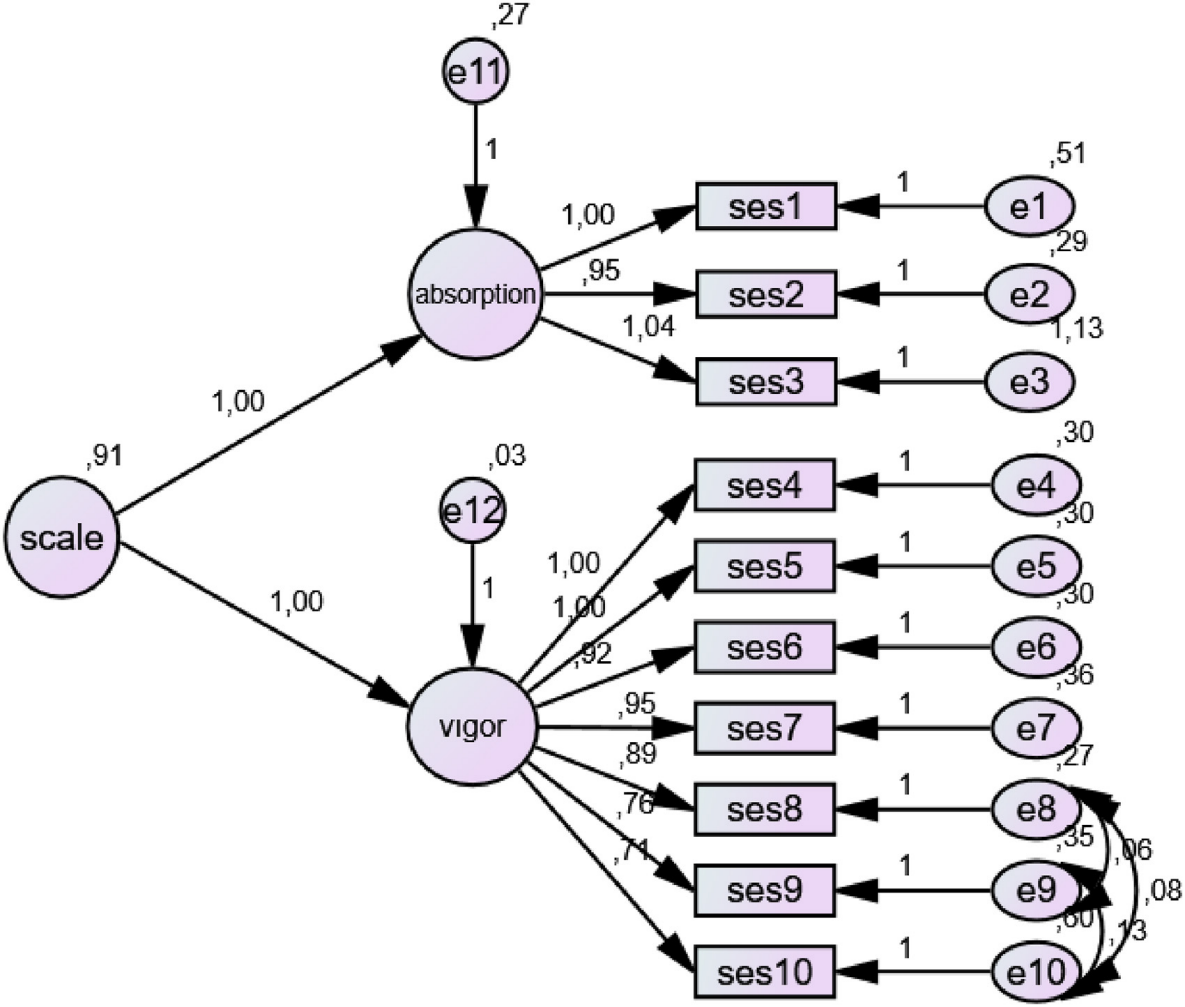


#### Perceived Social Support Scale – short form (F-SozU K-3)

The scale was originally developed by Petersen et al. (2024). Evidence for its use in Turkish samples, including confirmatory factor analysis and reliability assessment, was reported by Türk et al. (2025). The scale consists of three items rated on a five-point Likert-type scale, with no reverse-coded items. Total scores are obtained by summing item responses. In the original study, internal consistency reliability was reported as Cronbach’s α = 0.80. In the present study, the scale demonstrated acceptable internal consistency (Cronbach’s α = 0.73).


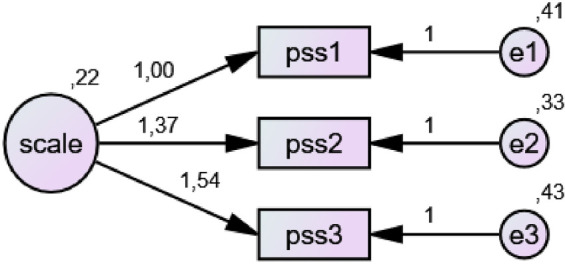


#### Validity and reliability analyses of the scales

To evaluate the construct validity of the scales used in the study, first- and second-order confirmatory factor analyses (CFA) were performed. Since the data met the assumption of multivariate normality, the Maximum Likelihood (ML) estimation method was employed in the analyses. In the CFA results, when deemed necessary to achieve acceptable fit indices commonly adopted in the literature, theoretically appropriate model modifications were applied in accordance with the conceptual framework. The model fit, reliability levels, and validity indicators of the scales used in the study were examined in detail and are presented in Table 1. Threshold values recommended by Byrne (2016), Gürbüz (2021a) were taken as reference for fit indices. For the Physical Activity Enjoyment Scale, values of X^2^/df = 7.715 and RMSEA = 0.095 were observed. Although these values exceed conventional cut-off criteria, Gürbüz (2021b) noted that in large samples (*n* > 700), it is possible for X^2^/df values to exceed 5. Similarly, MacCallum et al. (1996) stated that RMSEA values between 0.08 and 0.10 may be considered acceptable in large samples. Regarding perceived social support, it was reported that fit indices could not be calculated due to the saturated nature of the model and the small number of items (Kenny, 2024). Internal consistency reliability of the scales was evaluated using Cronbach’s alpha coefficient. George and Mallery (2019) classify alpha values above 0.80 as “good” and values above 0.90 as “excellent,” while Kalaycı (2018) stated that values between 0.60 and 0.80 indicate acceptable reliability. In evaluating construct validity, Average Variance Extracted (AVE) and Composite Reliability (CR) values were also considered. Fornell and Larcker (1981) indicated that AVE values should be above 0.50 and CR values should exceed 0.70. In addition, Maruf et al. (2021) suggested that AVE values above 0.40 may also be acceptable as long as CR values are at an adequate level. Findings related to these values, including CFA results for each scale, Cronbach’s alpha coefficients, skewness and kurtosis values, as well as AVE and CR values, are presented in the table.

**TABLE 1:** Fit indices and threshold values used in SEM, Cronbach’s alpha, normality analyses, Average Variance Extracted (AVE) and Composite Reliability (CR).

Index	Good fit	Acceptable fit	PACES	ICSS	SES	PSS
X^2^/df	<3	<3 (X^2^/df) < 5	7.715	3.370	4.543	–
GFI	>0.95	>0.90	0.957	0.992	0.964	–
CFI	>0.95	>0.90	0.974	0.997	0.982	–
RMSEA	<0.05	<0.08	0.095	0.056	0.069	–
SRMR	<0.05	<0.08	0.030	0.010	0.023	–
Cronbach alpha	0.90	0.60–0.80	0.932	0.943	0.940	0.73
Skewness	–	−3/+3	−1.706	−1.659	−1.147	−1.184
Kurtosis	–	−3/+3	2.357	2.163	0.749	0.506
AVE	>0.50	>0.40	0.640	0.757	0.705	0.480
CR	>0.70	>0.70	0.934	0.941	0.959	0.732

### Data analysis

Before conducting the main analyses, missing values and outliers in the dataset were examined using SPSS 25.0. At this stage, compliance with the assumption of multivariate normality was evaluated, and the relationships among the independent, dependent, mediating, and moderating variables were examined using Pearson correlation analysis. The assumption of normal distribution was tested based on Mahalanobis distances, Z-scores, and skewness and kurtosis values computed for the scale scores. Skewness and kurtosis coefficients within the ±3 range and Z values between −3 and +3 indicate that the data are consistent with a normal distribution (Kalaycı, 2018). Linear relationships among variables were examined through scatter plots, and no meaningful deviation was detected. To determine whether multicollinearity was present, tolerance and Variance Inflation Factor (VIF) values were examined. Tolerance values > 0.20 and VIF values < 10 indicated that there was no multicollinearity among the independent variables. Confirmatory factor analysis (CFA) was conducted using AMOS 21 to test the factor structures of the measurement instruments used in the study. After confirming the factor structures, bootstrap-based regression analyses were performed to examine the hypothesized relationships among variables (Hayes, 2022). Hypothesis testing was conducted using the PROCESS Macro developed by Hayes (2022). Model 4 was used to test the mediation effect, Model 1 was used to test the moderation effect, and Model 14 was used to test the moderated mediation effect. Within the regression analyses, the bootstrap resampling technique was performed using 5,000 bootstrap samples (Hayes et al., 2017). For the model results to be considered statistically significant, the 95% confidence interval values must not include zero (Hayes et al., 2017; Preacher and Hayes, 2008).

### Common method bias test

Two diagnostic approaches were used to evaluate the potential influence of common method bias (CMB). First, Harman’s single-factor test was conducted using Principal Axis Factoring on all measurement items. In order to assess whether the dataset contained a Common Method Variance problem, Harman’s single-factor test was applied by following the procedures recommended by Podsakoff et al. (2024). The results showed that, in the unrotated factor solution, a single factor explained 47.28% of the total variance. Considering the “majority of variance explained” principle stated by Podsakoff et al. (2024) and the commonly accepted 50% threshold value reported in the literature (Eichhorn, 2014), this value remained below the critical threshold. This finding indicates that no single factor was dominant in the dataset and that the threat of common method bias was minimal. Second, beyond the single-factor test, the correlation matrix of the latent constructs was examined as an additional diagnostic approach. The findings indicated that, although the correlations among variables were significant, the highest correlation was 0.665. This value is well below the high threshold (*r* > 0.90) that would indicate that common method variance constitutes a serious concern. Taken together, these diagnostic procedures provide converging evidence that common method variance is unlikely to constitute a serious threat to the validity of the findings in the present study.

## Results

### Correlation analysis and descriptive statistics

In this study, the correlations, means, and descriptive statistics among athletes’ PACES, ICSS, SES, and PSS scores are presented in Table 2.

**TABLE 2:** Correlations among variables and descriptive statistics.

Variables	PACES	ICSS	SES	PSS	*M*	SD
PACES (enjoyment)	–	–	–	–	52.04	5.994
ICSS (intention to continue)	0.471**	–	–	–	27.38	4.051
SES (sport engagement)	0.579**	0.665**	–	–	61.47	9.276
PSS (social support)	0.398**	0.334**	0.425**	–	13.36	2.122

*P < 0.05,

**p < 0.01.

An examination of the table indicates that there are statistically significant, positive relationships among PACES, ICSS, SES, and PSS. Specifically, moderate and statistically significant positive correlations were identified between PACES and ICSS (*r* = 0.471, *p* < 0.001), PACES and SES (*r* = 0.579, *p* < 0.001), PACES and PSS (*r* = 0.398, *p* < 0.001), ICSS and SES (*r* = 0.665, *p* < 0.001), ICSS and PSS (*r* = 0.334, *p* < 0.05), and SES and PSS (*r* = 0.425, *p* < 0.001).

### Mediation analysis (PROCESS Model 4)

To test the first hypothesis, a mediation model based on PROCESS Model 4 (PACES → SES → ICSS) was analyzed. Based on the bootstrap analyses, the indirect effect of enjoyment of physical activities on intention to continue sport participation through sport engagement was found to be statistically significant (*b* = 0.230, 95% CI [0.1848, 0.2799]). The variables included in the regression model were observed to explain approximately 45% of the total variance in intention to continue sport participation. These results indicate that the first hypothesis (H1) was supported.

### Moderation analysis (PROCESS Model 1)

To test the second hypothesis, a regression analysis based on PROCESS Model 1 was conducted to examine the moderation effect. The combined effects of sport engagement (X), perceived social support as the moderator (W), and the interaction term (X × W) on the outcome variable, intention to continue sport participation (Y), were evaluated. The findings revealed that the direct effect of sport engagement on intention to continue sport participation was statistically significant (*b* = 0.268, 95% CI [0.2417, 0.2950]). In contrast, the direct effect of perceived social support on intention to continue sport participation was not significant (*b* = 0.076, 95% CI [−0.0401, 0.1926]). Importantly, the interaction term representing the sport engagement × perceived social support interaction was statistically significant (*b* = −0.0147, 95% CI [−0.0245, −0.0049]), indicating that perceived social support moderated the relationship between sport engagement and intention to continue sport participation.

To further examine the nature of the moderation effect, a simple slopes analysis was conducted. The results indicated that the effect of sport engagement on intention to continue sport participation was significant when perceived social support was low (*b* = 0.303, 95% CI [0.2726, 0.3336]), moderate (*b* = 0.244, 95% CI [0.2099, 0.2787]), and high (*b* = 0.244, 95% CI [0.2099, 0.2787]). The conditional effects at the moderate and high levels of perceived social support were identical in the present sample, which is reflected in the PROCESS output and attributable to rounding of the bootstrapped estimates. These findings indicate that the second hypothesis (H2) was supported. The graphical representation of this moderation effect is presented below. These results suggest that perceived social support functions as a contextual boundary condition rather than a direct predictor of intention to continue sport participation. The graphical representation of this moderation effect is presented below.


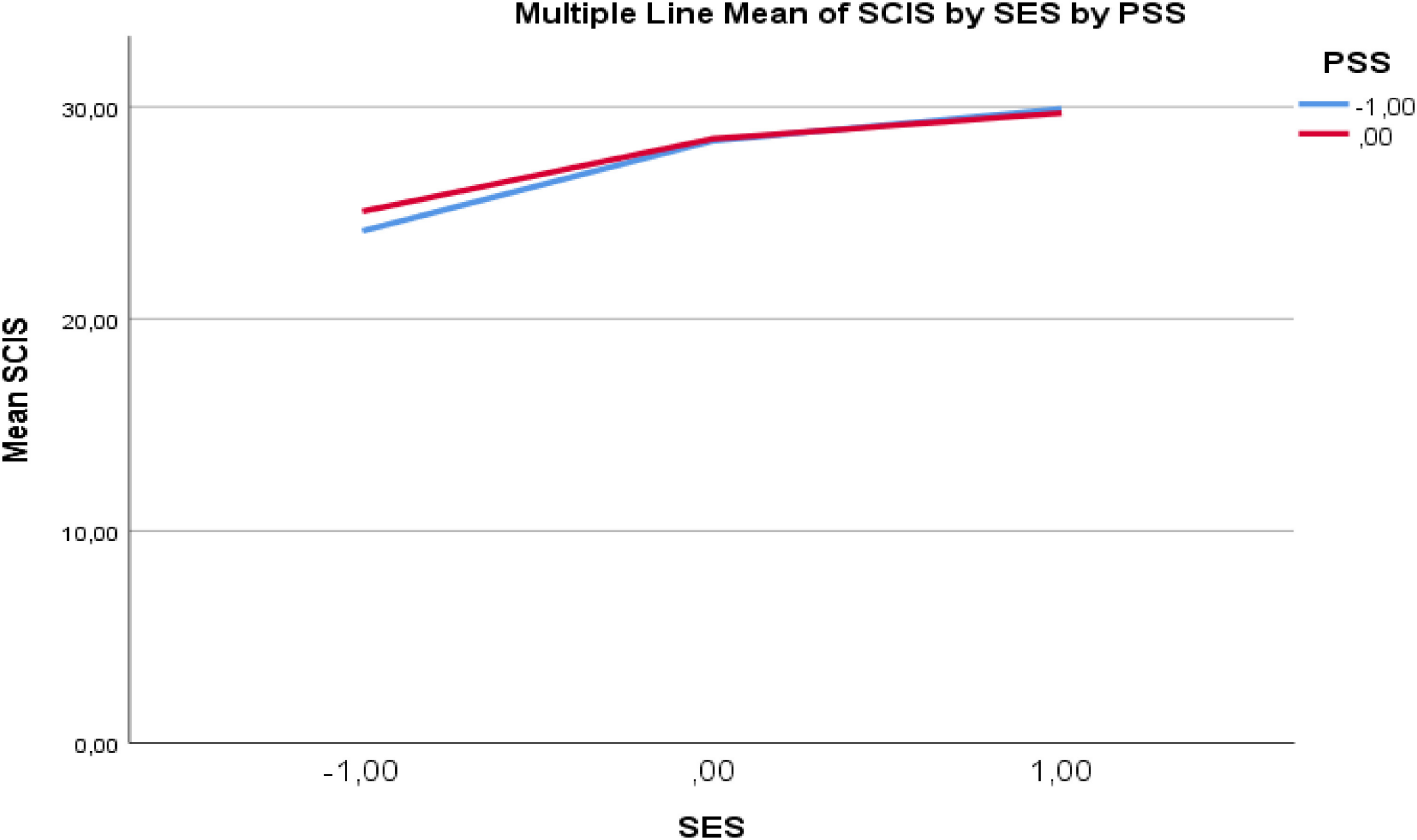


### Moderated mediation analysis (PROCESS Model 14)

To test the third hypothesis, PROCESS Model 14 was established, in which perceived social support assumed the role of a moderator within the indirect (mediated) relationship. The results of the moderated mediation analysis are presented in Table 3. The analysis conducted to determine whether perceived social support moderates the indirect effect of enjoyment of physical activities on intention to continue sport participation through sport engagement showed that the index of moderated mediation was significant (*b* = −0.010, 95% CI [−0.020, −0.0005]). This finding demonstrates that perceived social support plays a moderating role in the indirect effect. Accordingly, this result supports the third hypothesis (H3).

**TABLE 3:** Bootstrap regression analysis results.

Variables	SES			PACES			ICSS		
	*b*	LLCI	ULCI	*b*	LLCI	ULCI	*b*	LLCI	ULCI
Model 4 (H1)									
PACES (X)	0.895^**^	0.804	0.986	–	–	–	0.087**	0.043	0.131
SES (M)	–	–	–	–	–	–	0.257^**^	0.229	0.286
R^2^	0.334						0.452		
Bootstrap indirect effect	PACESSESICSS *b* = 0.230, %95 GA[0.184, 0.279]								
Model 1 (H2)									
SES (X)	–	–	–	–	–	–	0.268**	0.241	0.295
PSS (W)	–	–	–	–	–	–	0.076	−0.040	0.192
X*W (interaction)	–	–	–	–	–	–	−0.0147**	−0.024	−0.004
R^2^							0.451		
Model 14 (H3)									
PACES (X)	–	–	–	–	–	–	0.070**	0.0242	0.116
SES (M)	–	–	–	–	–	–	0.247**	0.218	0.277
PSS (W)	–	–	–	–	–	–	0.049	−0.068	0.166
X*W (interaction)	–	–	–	–	–	–	−0.011*	−0.021	−0.001
R^2^							0.457		
Indirect effect	*b*	LLCI	ULCI						
Low PSS	0.246**	0.195	0.299	–	–	–	–	–	–
Medium PSS	0.205**	0.154	0.257	–	–	–	–	–	–
High PSS	0.205**	0.154	0.257	–	–	–	–	–	–
Moderated mediation index	−0.010**	−0.020	−0.0005	–	–	–	–	–	–

*p < 0.05,

**p < 0.01.

Based on the simple slopes analysis, the indirect effects of enjoyment of physical activities on intention to continue sport participation through sport engagement were statistically significant when perceived social support was low (*b* = 0.2464, 95% CI [0.1953, 0.2997]), moderate (*b* = 0.2051, 95% CI [0.1542, 0.2573]), and high (*b* = 0.2051, 95% CI [0.1542, 0.2573]). Overall, the indirect effect remained significant across low, medium, and high levels of perceived social support; however, as perceived social support increased, the effect decreased up to a certain point and then remained stable.


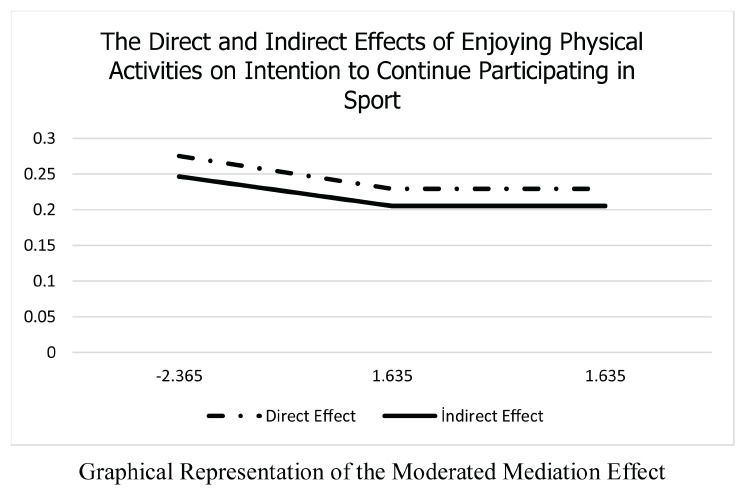


## Discussion

This study examined the effect of athletes’ enjoyment of physical activities on their intention to continue sport participation, as well as the mediating role of sport engagement and the moderating role of perceived social support in this relationship. The findings indicated that enjoyment of physical activities significantly increased intention to continue sport participation through sport engagement and that this relationship was moderated by perceived social support. These results are consistent with the theoretical framework regarding the interaction between psychological and social factors in sport and physical activity participation.

Enjoyment of physical activities is a strong motivational determinant of individuals’ sport participation. Pleasure and happiness derived from physical activity play an important role in the development of behavioral engagement and the intention to continue participating (Yang et al., 2025). Similarly, it has been reported that enjoyment derived from sport is positively associated with individuals’ intention to continue sport participation and that social identity strengthens enjoyment perceptions (Eskiler and Kaymakçı, 2025). Within the context of Self-Determination Theory and intrinsic motivation approaches, these findings suggest that enjoyment derived from physical activity enhances behavioral engagement and intentions. In addition, the sport psychology literature highlights the influence of the social environment on participation behaviors. Social support functions not only as an emotional resource but also as a key environmental factor enabling individuals to sustain sport participation behaviors (Yücekaya et al., 2025). The findings of the present study show that perceived social support shapes intention to continue sport participation through both direct and indirect pathways, underscoring the importance of social support research in the context of physical activity participation and continuity.

The findings demonstrated that the indirect effect of enjoyment of physical activities (PACES) on intention to continue sport participation (ICSS) through sport engagement (SES) was significant (*b* = 0.230, 95% CI [0.184, 0.279]). This result is consistent with theoretical approaches suggesting that intrinsic components of motivation influence behavioral engagement and the process of sustained participation in physical activity contexts. In particular, recent studies grounded in Self-Determination Theory indicate that enjoyment, satisfaction of psychological needs, and intrinsic motivation increase participation in physical activity (Barbosa Cano and Gomez-Baya, 2025; Teixeira et al., 2022). Enjoyment derived from physical activity represents a psychological experience indicating that individuals’ basic psychological needs are being satisfied, and the satisfaction of these needs triggers intrinsic motivational processes that support sport engagement and intention to continue sport participation (Ryan and Deci, 2017; Tao and Yu, 2025; Zorba, 2024). This mechanism also suggests that enjoyment of physical activity may constitute a direct psychological source of strength for maintaining behavior. One study reported that perceived enjoyment was positively associated with exercise engagement and behavioral intention (Joung et al., 2024). Similarly, research conducted with adults has shown that enjoyment derived from physical activity has important effects on exercise habit, intention to maintain exercise, and exercise frequency (Sun et al., 2025; Teixeira et al., 2022). These findings indicate that enjoyment and motivational processes strengthen the relationship between behavioral engagement and intention to continue. A study conducted with university athletes also showed that enjoyment derived from sport was positively associated with intention to continue sport participation and influenced behavioral intention together with the social context (Eskiler and Kaymakçı, 2025). Furthermore, the Intention to Continue Sport Scale developed by Özkurt et al. (2025) suggests that this intention is closely related to emotional and motivational components and that enjoyment-based motivation plays an important role in behavioral outcomes. It is also frequently reported that enjoyment derived from sport shapes sport engagement and intention to continue not only through individual motivation but also through its associations with social context, group identity, and environmental factors (Mo et al., 2024; Sañudo et al., 2024). Overall, these findings demonstrate that our study is theoretically supported from both individual and social psychology perspectives. In sum, the support for H1 indicates that enjoyment of physical activities represents an intrinsic psychological resource that enhances sport engagement, which in turn positively shapes intention to continue sport participation. This conclusion strongly aligns with current theoretical models and empirical findings explaining the link between motivational processes and behavioral outcomes.

The findings also showed that the direct effect of sport engagement (SES) on intention to continue sport participation (ICSS) was significant (*b* = 0.268, 95% CI [0.242, 0.295]). In contrast, the direct effect of perceived social support (PSS) on intention to continue sport participation was not significant (*b* = 0.076, 95% CI [−0.040, 0.198]), whereas the interaction term between sport engagement and intention to continue sport participation was significant (*b* = −0.0147, 95% CI [−0.0245, −0.0049]). This indicates that social support moderates this relationship. This finding can be explained within the frameworks of Social Cognitive Theory (Bandura, 1986) and Self-Determination Theory (Ryan and Deci, 2020). According to Social Cognitive Theory, individuals’ behavioral intentions and engagement may be shaped by environmental factors and perceived support. Self-Determination Theory proposes a strong association between social support and satisfaction of psychological needs, which may strengthen intrinsic motivation and behavioral engagement. In this context, whether social support is low or high may influence the direction and magnitude of the relationship between sport engagement and intention to continue sport participation. Yuan et al. (2024) similarly emphasized that social support level affects athletes’ motivational engagement and behavioral intentions. These studies show that social support plays a critical role in the interaction between individual motivation and behavioral intention. Sport participation is not only a domain in which physical abilities are displayed but also a dynamic process involving intensive social interactions. In this process, athletes’ motivation and continuity should not be evaluated independently of their social ties. Moreover, the potential of the social context to strengthen or weaken sport engagement can be explained through factors such as group identity, peer support, and coach–athlete interactions in sport settings (Graupensperger et al., 2020; Stevens et al., 2017). Research suggests that these factors are determinative for athletes’ psychological functioning. In particular, perceived and received support from coaches directly shapes athletes’ self-confidence and psychological wellbeing, thereby increasing dedication to sport and continuity (Coussens et al., 2025). Similarly, a strong social identity and a sense of “we” within the team environment nurture athletes’ sense of belonging, prevent disengagement from sport, and support subjective wellbeing (Graupensperger et al., 2020). Therefore, for sustainable sport habits, the presence of a supportive social climate is critically important alongside technical skills. These findings show that sport engagement does not always lead directly to intention to continue sport participation and that environmental factors such as social support can regulate this process. This negative interaction may be interpreted within a compensatory framework. When perceived social support is low, sport engagement may function as a stronger internal motivational resource compensating for the lack of environmental support. Conversely, when social support is high, external motivational resources may partially substitute for individual engagement, resulting in a relatively weaker slope. This pattern suggests that perceived social support may operate as a contextual boundary condition rather than a simple amplifying factor. Consequently, the H2 hypothesis was supported. The results indicate that the increasing effect of sport engagement on intention to continue sport participation varies depending on the level of perceived social support. This reveals, both theoretically and practically, that athletes’ motivational and social contexts shape their intention to continue sport participation.

Finally, the findings showed that the indirect effect of enjoyment of physical activities (PACES) on intention to continue sport participation (ICSS) through sport engagement (SES) was significantly moderated by perceived social support (PSS) (*b* = −0.010, 95% CI [−0.020, −0.0005]). While the slope analyses demonstrated that the indirect effect was significant at low, moderate, and high levels of social support, the effect decreased up to a certain point as social support increased and then remained stable. This pattern suggests that social support functions not as an unlimited enhancer in sport settings but rather as a foundational psychological ground required for sustainable participation. Zhang and Qian (2022) define participation as a stable, continuous sport experience encompassing individuals’ positive emotions and perceptions toward sport within a broader framework. In this context, enjoyment derived from physical activity plays a critical role in individuals’ ability to sustain long-term sport participation and integrate such activities into daily life routines (de la Torre-Cruz et al., 2024; Ferrando-Terradez et al., 2025). From a theoretical perspective, these results are strongly consistent with the basic assumptions of Self-Determination Theory (SDT). According to SDT, autonomy-supportive behaviors from coaches and peers satisfy individuals’ need for relatedness and enable the internalization of external factors (Ryan and Deci, 2020). Comprehensive meta-analyses have demonstrated that perceived support from the social environment is one of the strongest external factors predicting not only immediate enjoyment but also long-term behavioral engagement and mental wellbeing (Vasconcellos et al., 2020). Particularly for an intrinsic feeling such as enjoyment to transform into a stable intention over time, the facilitating role of the social context is crucial. Peng et al. (2025) also noted that post-exercise enjoyment is an important factor influencing outcomes. From the perspective of social psychological models, the findings explain how the intention–behavior gap can be bridged through social factors (Hagger et al., 2020). Social support can be conceptualized as an environmental feedback mechanism that instills a sense of “I can do it” and translates intention into action. However, the “plateauing” finding in the present study indicates that social support alone is not sufficient; beyond a certain threshold, individuals’ internal engagement processes (e.g., group identity, intrinsic satisfaction) become more influential and external support assumes a complementary role (Graupensperger et al., 2020). In addition, Kinoshita et al. (2023) highlighted the critical roles of parents and coaches in enabling young people to experience positive psychological experiences in sport, emphasizing that each stakeholder has an influence on individuals’ positive psychological development (intention to continue sport participation and subjective wellbeing). Overall, social support functions as a facilitator of the psychological process that converts enjoyment derived from sport into a concrete intention to continue. Consistent with previous findings using moderated mediation frameworks in physical activity contexts, the present results further support the view that psychological and behavioral outcomes may operate through conditional indirect effects (Seçer et al., 2025). In this respect, the presence of social support networks in sport settings strengthens its position in literature as a strategic resource that supports sustained engagement, beyond serving as an emotional comfort zone. Consequently, the H3 hypothesis was strongly supported. The findings indicate that perceived social support is a critical factor moderating the pathway from enjoyment of physical activities to intention to continue sport participation through sport engagement, and that it plays a central role in both individual and social psychological processes.

## Strengths, limitations, and future directions

### Strengths

One of the primary strengths of this study is that it integrates multiple theoretical perspectives into a comprehensive model to explain athletes’ intention to continue sport participation. The research model was constructed based on Self-Determination Theory, the sport engagement framework, the Theory of Planned Behavior, Social Cognitive Theory, and Ecological Systems Theory. This theoretical integration demonstrates that intention to continue sport participation is shaped not only by individual motivation but also by psychological engagement and interactions with the social environment. Another strength of the study is that motivational and behavioral processes were tested together with mediating and moderating mechanisms. Demonstrating that the effect of enjoyment of physical activities on intention to continue sport participation operates through sport engagement, and that this indirect effect varies depending on the level of perceived social support, highlights the dynamic and contextual nature of sport participation. This approach provides an advanced analytical framework for explaining sport continuation behavior. From a methodological standpoint, the large sample size constitutes a major advantage. The high number of participants increased the statistical power of the analyses and supported the robustness of the findings. In addition, the use of measurement instruments with strong validity and reliability, testing measurement structures through confirmatory factor analyses, and controlling for common method bias are among the methodological features that strengthen the rigor of the study. Finally, by addressing intention to continue sport participation not only through direct effects but also within the context of indirect and conditional effects, the study contributes to a deeper understanding of motivational processes in sport psychology. In this respect, the study offers a strong structure both theoretically and practically.

### Limitations

This study has several limitations. First, the cross-sectional design restricts causal interpretations of the relationships among variables. Although conditional process analyses were conducted, the data structure does not permit definitive cause–effect conclusions. Therefore, the findings should be interpreted as associative rather than causal. The findings are correlational in nature and do not directly reveal changes over time or establish cause–effect relationships. Second, the reliance on self-report data introduces the risk of perception-based bias. Social desirability tendencies or individual differences in perception may lead to deviations between reported scores and actual levels of the measured constructs. Another limitation is that perceived social support was addressed as a general construct. Considering that social support may exert different effects depending on its source, the absence of such differentiation in the current study may constrain the interpretation of the findings to some extent. In addition, because the sample consisted only of active athletes, generalizing the results to non-athletes, recreational participants, or individuals who are new to sport participation is limited. Although the Perceived Social Support Scale used in this study consists of only three items, it has been validated in recent research and demonstrated acceptable psychometric properties. However, the brevity of the instrument may limit the comprehensiveness of the construct measurement. Future studies are encouraged to employ multidimensional sport-specific social support scales to capture different sources and types of support in greater detail.

### Future directions

Future research is encouraged to employ longitudinal or experimental designs to clarify the relationships among enjoyment of physical activities, sport engagement, and intention to continue sport participation more precisely. Such designs would make important contributions to explaining the temporal dynamics and causal structure of motivational processes. Moreover, examining social support separately according to its source (e.g., family, friends, teammates, coaches) would allow a more detailed understanding of distinct social influence mechanisms on intention to continue sport participation. This may also support the development of more targeted intervention programs in applied settings. Future studies may further contribute to a more comprehensive explanation of intention to continue sport participation by incorporating additional psychological variables into the model, such as resilience, mindfulness, motivational climate, or athletic identity. Finally, testing the model across different age groups, sport branches, and cultural contexts would enhance the generalizability of the findings.

## Conclusion

This study revealed the joint and interactive roles of enjoyment of physical activities, sport engagement, and perceived social support in the formation of athletes’ intention to continue sport participation. The findings indicate that enjoyment derived from physical activity strengthens sport engagement, and that this engagement is meaningfully reflected in intention to continue sport participation. Moreover, perceived social support regulates this process, emphasizing the contextual nature of motivational and behavioral dynamics. The results suggest that intention to continue sport participation cannot be explained solely through individual motivation; rather, it represents a multidimensional structure shaped through interaction with psychological engagement and the social environment. Accordingly, in practices aimed at increasing participation continuity in sport settings, enhancing athletes’ enjoyment of physical activity, strengthening their sport engagement, and fostering supportive social environments may be considered important strategies. Overall, the present study offers a theoretically grounded and practice-oriented model explaining intention to continue sport participation, and it underscores the importance of addressing motivational processes together with the social context within the field of sport psychology.

## Data Availability

The raw data supporting the conclusions of this article will be made available by the authors, without undue reservation.
